# The Value of Physiological Scoring Criteria in Predicting the In-Hospital Mortality of Acute Patients; a Systematic Review and Meta-Analysis

**DOI:** 10.22037/aaem.v9i1.1274

**Published:** 2021-09-09

**Authors:** Amirmohammad Toloui, Arian Madani Neishaboori, Seyedeh Niloufar Rafiei Alavi, Mohammed I M Gubari, Amirali Zareie Shab Khaneh, Maryam Karimi Ghahfarokhi, Fatemeh Amraei, Zahra Behroozi, Mostafa Hosseini, Sajjad Ahmadi, Mahmoud Yousefifard

**Affiliations:** 1 Physiology Research Center, Iran University of Medical Sciences, Tehran, Iran.; 2 Community Medicine, College of Medicine, University of Sulaimani, Sulaimani, Iraq.; 3 Department of Epidemiology and Biostatistics, School of Public Health, Tehran University of Medical Sciences, Tehran, Iran.; 4 Emergency Medicine Research Team, Faculty of Medicine, Tabriz University of Medical Sciences, Tabriz, Iran.; 5 Department of Physiology, School of Medicine, Iran University of Medical Sciences, Tehran, Iran.; 6 Department of Emergency Medicine, Tabriz University of Medical Sciences, Tabriz, Iran.; a First and second authors have contributed equally

**Keywords:** Clinical decision rules, Wounds and injuries, Mortality, Predictive value of tests

## Abstract

**Introduction:**

There is no comprehensive meta-analysis on the value of physiological scoring systems in predicting the mortality of critically ill patients. Therefore, the present study intended to conduct a systematic review and meta-analysis to collect the available clinical evidence on the value of physiological scoring systems in predicting the in-hospital mortality of acute patients.

**Method:**

An extensive search was performed on Medline, Embase, Scopus, and Web of Science databases until the end of year 2020. Physiological models included Rapid Acute Physiology Score (RAPS), Rapid Emergency Medicine Score (REMS), modified REMS (mREMS), and Worthing Physiological Score (WPS). Finally, the data were summarized and the findings were presented as summary receiver operating characteristics (SROC), sensitivity, specificity and diagnostic odds ratio (DOR).

**Results:**

Data from 25 articles were included. The overall analysis showed that the area under the SROC curve of REMS, RAPS, mREMS, and WPS criteria were 0.83 (95% CI: 0.79-0.86), 0.89 (95% CI: 0.86-0.92), 0.64 (95% CI: 0.60-0.68) and 0.86 (95% CI: 0.83-0.89), respectively. DOR for REMS, RAPS, mREMS and WPS models were 11 (95% CI: 8-16), 13 (95% CI: 4-41), 2 (95% CI: 2-4) and 17 (95% CI: 5-59) respectively. When analyses were limited to trauma patients, the DOR of the REMS and RAPS models were 112 and 431, respectively. Due to the lack of sufficient studies, it was not possible to limit the analyses for mREMS and WPS.

**Conclusion:**

The findings of the present study showed that three models of RAPS, REMS and WPS have a high predictive value for in-hospital mortality. In addition, the value of these models in trauma patients is much higher than other patient settings.

## 1. Introduction:

Trauma is one of the most important causes of mortality and disability in societies, especially in developing countries (1). Statistics show that trauma and accidents is the third leading cause of death in the entire population of Iran and is unfortunately the leading cause of death among young people (2). Since the young population constitutes the majority of casualties, the burden of trauma and accidents is far greater than many infectious and non-communicable diseases. The extent of the problem is such that, according to the World Health Organization, up to 50% of people who are hospitalized due to unintentional accidents are discharged with some form of disability (3).

Studies show that if the severity of trauma and injury is diagnosed quickly, the mortality rate and the resulting disability will be significantly reduced (4). For this purpose, diagnostic modalities such as CT scan, magnetic resonance imaging, ultrasound, and chest x-ray are used to identify the severity of injury in the clinic. However, for reasons such as the lack of proper access to this equipment in many cases, the risks of exposure to radiation, as well as the limitations of these diagnostic tests (for instance the low diagnostic value of chest X-ray in identifying pneumothorax, the value of ultrasound depending on the skill of the operator and etc.) Researchers have long sought other ways to classify patients. One of these methods is the use of scoring models based on clinical examinations. These models, known as scoring systems, have been in research for decades and have been gradually modified. However, the use of these models has always been associated with disadvantages and limitations (5). For example, the calculation of many of the introduced models and their scoring methods are complex and, in some cases, their validity has not been examined in different clinical conditions. Therefore, research in this field is still ongoing and a number of new models have been presented lately.

In recent years, health departments have proposed the establishment of physiological scoring systems to identify patients at high risk for mortality so that the management of these trauma patients can be more well-structured, thereby reducing the burden of trauma (6). Based on this, several physiological scoring systems were developed and provided to researchers, such as early-warning scoring system (7-10), Worthing Physiological Scoring System (WPSS) (11) Rapid Emergency Medicine Score (REMS), Acute Physiology and Chronic Health Evaluation (APACHE) II, and Revised Trauma Score. Nevertheless, there is still no definitive conclusion as to whether the use of physiological scoring systems can reliably predict the outcome of trauma patients. One of the ways to answer such a question is to conduct a systematic review and meta-analysis on the matter. In this regard, a meta-analysis conducted on poisoning patients from 29 studies in 2017 showed that the APACHE II score in deceased patients was significantly lower than in living patients. The best cut-off point for APACHE II was 10, at which the cut-off had a sensitivity of 88% and a specificity of 84% (12). Another meta-analysis by Hamilton et al. In 2018 examined the diagnostic value of early warning scoring system in septic patients. In this analysis, which was performed on 6 studies, it was found that this scoring system cannot accurately predict the mortality of patients with sepsis (13). However, research in this field is still open and systematic reviews are being conducted (14, 15).

Although many studies have been performed in this field in recent years, a comprehensive meta-analysis has not yet been performed on other physiological scoring systems (16-19). Based on this, the researchers of the present study intended to conduct a systematic review and meta-analysis to collect the available clinical evidence on the value of physiological scoring criteria in predicting the in-hospital mortality of patients. The studied physiological criteria included Rapid Acute Physiology Score (RAPS), Rapid Emergency Medicine Score (REMS), modified REMS (mREMS), and Worthing Physiological Score (WPS). Although the initial objective of the present study focused only on trauma patients, in the end, in addition to studies performed on trauma patients, other causes of acute hospitalization including infection and sepsis were also included.

## 2. Methods:

**Study design**

The aim of this study was to evaluate the value of physiological scoring models in predicting the in-hospital outcomes of acute hospitalized patients. In the present study, the MOOSE guideline was used, which is a guide for performing systematic review and meta-analysis in observational research (20).

**2.1. Definition of PICO**

The problem or population studied (P) includes human studies performed on acute hospitalized patients. Index (I) is the physiological scoring models including RAPS, REMS, mREMS and WPS. Comparisons (C) are made with the living group and the assessed outcome (O) is patients’ mortality.

**2.2. Search strategy**

To achieve the objectives of the present study, an extensive search was conducted in electronic databases and related article sources. Grey literature search was another strategy that was undertaken in the present project.

The search of electronic databases was carried out systematically under the supervision of an expert and researcher in the field of systematic review. At this stage, related keywords were selected using MeSH and Emtree databases, consultation with experts and search in the title and abstract of related articles. The search strategy for each database was then defined using the site’s guideline of search strategy. The approach on how to search and summarize the data has been reported in the previous meta-analyses of the present study’s researchers (21-30). The Medline, Embase, Web of Science, and Scopus databases were searched until the end of year 2020. Search query in Medline is presented below.

*(rapid emergency medicine score[tiab] OR worthing physiological scoring system[tiab] OR Rapid Emergency Medical Score[tiab] OR Rapid Acute Physiology Score[tiab] OR Physiology scoring system[tiab])*

**2.3. Selection criteria**

Human diagnostic studies performed to assess the value of physiological scoring models and their predictive power regarding patients’ outcomes were included. The study population weres human studies with no age, sex, or racial restrictions.

Case report studies, case series, review articles, failure to evaluate index test compared to the standard reference, and not following up patients until their discharge from the hospital were our exclusion criteria.

 2.4. **Data extraction **

Screening and summarizing of articles, and entering their data into the checklist, as well as the final quality control were executed by two independent researchers. Any disagreement was resolved through discussion with a third researcher. Articles were summarized based on a checklist designed according to the PRISMA statement guidelines (31). The extracted data included information related to the study design, sample characteristics (age, sex, mechanism of injury), number of samples examined, outcome, and possible biases (Bias). If two or more articles were based on the same dataset, the study with the largest sample size or the longest follow-up time was included. If the required data was not provided in the article, the data was requested by contacting the corresponding author. If data were recorded separately for different subgroups (such as sex or age, etc.), they were entered in our study in the same way.

**2.5. Risk of bias of articles**

The quality was assessed using QUADAS-2 instructions (32). To evaluate the agreement between the two researchers, inter-rater reliability was examined in the qualitative evaluation of the studies. In case of disagreement, the dispute was resolved through discussion with a third researcher.

**2.6. Statistical analyses**

Analyses were performed using STATA 14.0 statistical program. All studies were summarized and categorized based on patients' outcomes (dead or alive) and true positives, true negatives, false positives, and false negatives were recorded accordingly. In the above-mentioned statistical program, analyses were performed using the “*midas*” command. Based on different sub-commands, the area under the curve (SROC) of each of the scoring models, their sensitivity, specificity, and diagnostic odds ratio (DOR) with 95% confidence interval (95% CI) were calculated. Based on the presence or absence of heterogeneity, a random effect model or a fixed effect model was used to perform the analyses, respectively. I2 test was used to evaluate the heterogeneity between studies. In cases of heterogeneity, meta-regression and subgroup analysis were performed to determine the cause of heterogeneity. Finally, the results of the studies were pooled and an overall effect size was presented. Deek’s Funnel Plot was used to identify publication bias (33). 

## 3. Results:

**3.1. Characteristics of the included studies**

**Figure 1:**
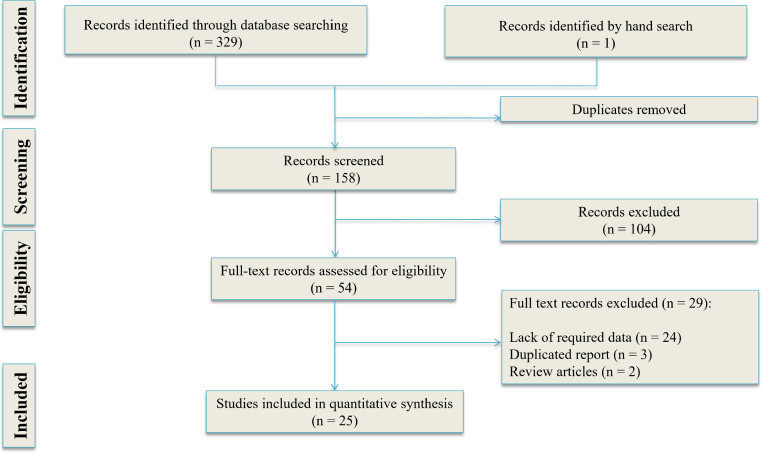
PRISMA flow diagram of the present study

Our search yielded 158 non-duplicate articles. Of these, 77 potentially eligible articles were studied in more detail and finally, 25 articles were included in the present meta-analysis (66-42) (Figure 1). There were 11 prospective cohort studies, 11 retrospective cohort studies, 1 case-control study, and 2 cross-sectional studies. These studies included 737,351 patients (47.16% male) and of all patients, 23,149 (3.14%) died. There were 6 studies on trauma patients, 9 studies on sepsis / infection patients, 5 studies on all acute conditions (mixed population) and 5 studies on non-trauma patients. Table 1 shows the characteristics of the included studies.

**3.2. Meta-analysis**

**The diagnostic value of REMS in predicting the in-hospital mortality**

**Figure 2:**
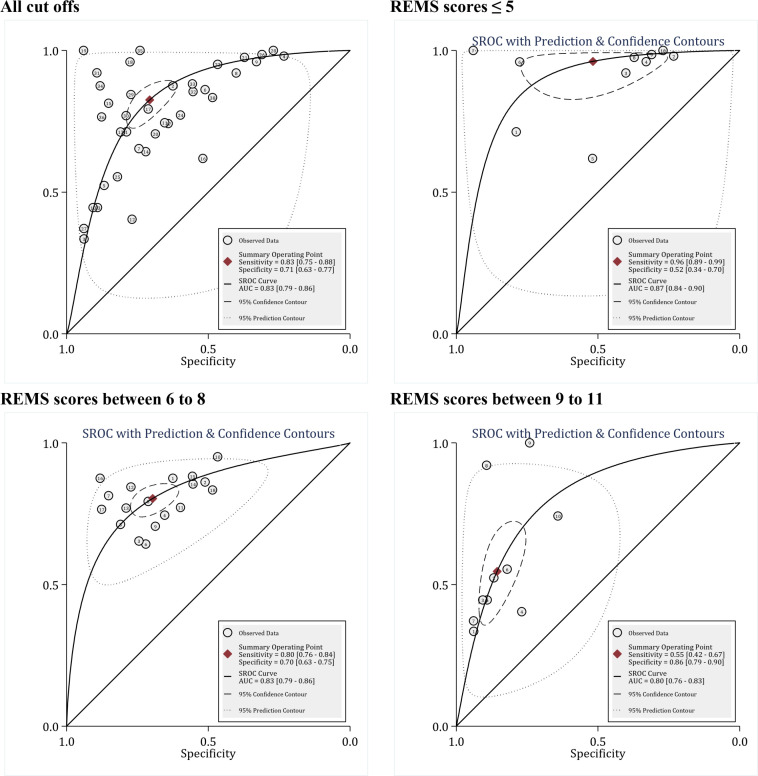
Prognostic value of Rapid Acute Physiology Score (REMS) in prediction of in-hospital mortality. SROC: Summary receiver operating characteristics; AUC: area under the curve

21 articles were included in the evaluation of the diagnostic value of REMS in predicting the in-hospital mortality of patients. These 21 articles contained 38 separate analyses in terms of different cut-off points. The total number of patients in these 21 studies was 578,373, of whom 10,862 died. The cut-off points for this model varied between 3 and 11. Overall analysis showed that the area under the ROC curve, regardless of the cut-off points, was 0.83 (95% CI: 0.79 to 0.86). Overall sensitivity, specificity, and DOR of REMS model in predicting the in-hospital mortality were 0.83 (95% CI 0.75 to 0.88), 0.71 (95% CI: 0.63 to 0.77), and 11 (95% CI: 8 to 16), respectively (Figure 2-A). Nevertheless, there was significant heterogeneity among studies (I2 = 100.0%); therefore, meta-regression was performed. Meta-regression showed that the most important sources of heterogeneity between studies were using different cut-off points, the difference in study design (retrospective and prospective), and different settings of patients (Table 2). Stratification of analysis based on these differences between studies caused a significant reduction in heterogeneity to the point that I2 was zero in some subgroups. Accordingly, the findings were reported separately for these subgroups.

The first and the most important factor influencing the prognostic value of REMS in predicting the in-hospital mortality was the different cut-off points between studies. Based on meta-regression, the cut-off points were divided into three groups: REMS scores≤5 (categories with sensitivity higher than 90%), REMS scores between 6 to 8 (categories with sensitivity between than 70% to 89%) and REMS scores≥8 (categories with sensitivity lower than 70%). The area under the ROC curve of the REMS model at the cut-off scores≤5, 5 to 8, and ≥8 was 0.87 (95% CI: 0.84 to 0.90), 0.83 (95% CI: 0.79 to 0.86), and 0.80 (0.76 to. 0.83), respectively (Figure 2-B to 2-D). In the evaluation of DOR between subgroups, it was found that the classification of patients based on REMS≤5 cut-off point had more clinical value than other cut-offs; since in this cut-off, DOR of REMS was 27 in predicting the in-hospital mortality, which was way more than cut-offs between 6 to 8 (DOR = 9) and ≥9 (DOR = 7) (Table 3).

In evaluating the role of difference in the type of study, it was found that 36 analyses had cohort design, while 1 study had a case-control design, and 1 had a cross-sectional design. Therefore, subgroup analysis was not useful for this factor. Another point obtained in subgroup analysis was the role of study design (retrospective versus prospective) on the predictive value of REMS. As Table 3 shows, the DOR of REMS was 15 in prospective studies and 9 in retrospective studies.

The setting of patients in the included studies was another factor influencing the findings on the predictive value of REMS. In this section, 4 analyses were performed on trauma patients, 9 analyses were performed on patients with sepsis / infection, 4 analyses were performed on non-trauma acute surgery, and 21 analyses were performed on all acute conditions. The interesting point was the very high prognostic value of REMS in trauma patients. DOR of REMS was 112 in predicting the in-hospital mortality of trauma patients, while in other patient settings the DOR value was much lower (DOR = 9 in sepsis / infection, DOR = 20 in non-trauma setting, and DOR = 8 in all acute settings) (Table 3).

**The diagnostic value of RAPS in predicting the in-hospital mortality**

In the evaluation of the diagnostic value of RAPS in predicting the in-hospital mortality, 8 articles were included, which included 12 separate analyses in terms of different cut-off points. The total number of patients in these 8 studies was 55052 patients, of which 710 patients died. The cut-off points presented for this model in the studies varied between 2 and 8.

**Figure 3:**
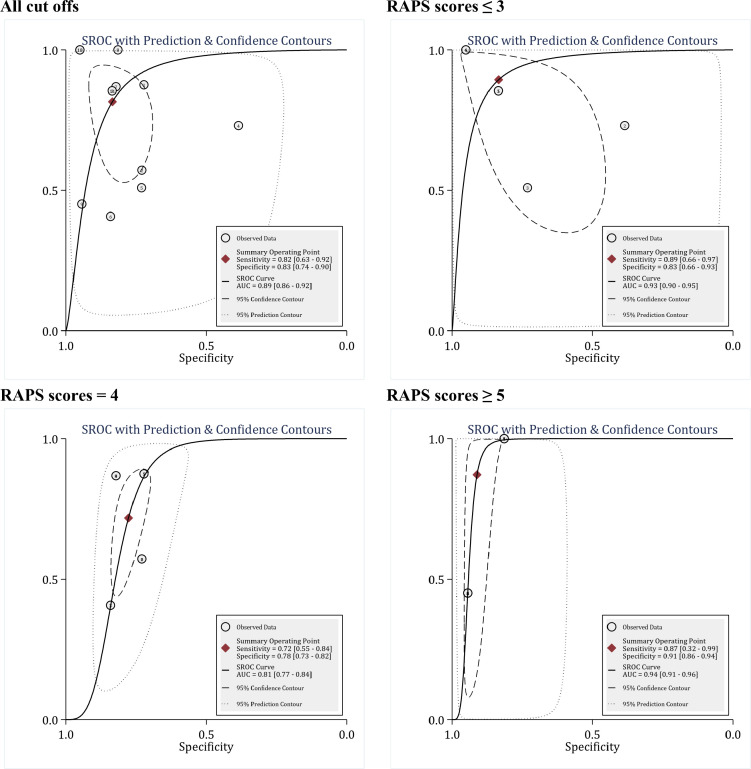
Prognostic value of Rapid Acute Physiology Score (RAPS)in prediction of in-hospital mortality. SROC: Summary receiver operating characteristics; AUC: area under the curve

The area under the ROC curve of RAPS in predicting the in-hospital mortality without considering the cut-off points was 0.89 (95% CI: 0.86 to 0.92) (Figure 3-A). The sensitivity, specificity, and overall DOR of RAPS in predicting the in-hospital mortality were 0.82 (95% CI 0.63 to 0.92), 0.83 (95% CI: 0.74 to 0.90) and 13 (95% CI: 4 to 41), respectively. However, there was significant heterogeneity between studies (I2 = 100.0%). In order to find the source of heterogeneity, meta-regression analysis was performed. Meta-regression showed that similar to REMS, the most important source of heterogeneity between studies in RAPS analysis was the use of different cut-off points, differences in study design (retrospective and prospective), type of study (cohort, case-control and cross-sectional), and different patient settings (Table 2). Stratification of analyses based on these differences between studies caused a significant reduction in heterogeneity, to the point where I2 was equal to zero in some subgroups. Accordingly, the findings were reported separately for these subgroups.

The first and most important factor influencing the prognostic value of RAPS in predicting the in-hospital mortality was the different cut-off points used between studies. In this section, RAPS cut-off points were divided into three groups: RAPS scores ≤3, RAPS score equal to 4, and RAPS scores of 7 to 8. The area under the ROC curve of the RAPS model at the cut-off points ≤3, 4, and 7 to 8 were equal to 0.93 (95% CI: 0.90 to 0.95), 0.81 (95% CI: 0.77 to 0.84), and 0.94 (0.91 to 0.96), respectively (Figure 3-B to 3-D). In the study of DOR between subgroups, it was found that the classification of patients based on RAPS scores 7 to 8 had a higher clinical value than other cut-off points, since the DOR of RAPS was 69 times higher in predicting the in-hospital mortality, which is much higher than when cut-off points 4 (DOR = 9) and ≤3 (DOR = 42) are used (Table 4).

In examining the role of difference in the type of study on the predictive value of RAPS, it was found that 8 of the 10 analyses were performed as cohort studies. Therefore, subgroup analysis was not very useful for this factor. In addition, the effect of difference in the design of study (retrospective versus prospective) on the predictive value of RAPS was not significant. As Table 4 shows, the DOR of RAPS was 17 in prospective studies and 13 in retrospective studies, both of which indicate a high clinical value for RAPS in outcome prediction.

The setting of patients in the included studies was another factor that caused heterogeneity in the findings of the RAPS section. In this part, 4 analyses were performed on trauma patients, 2 analyses were performed on patients with sepsis/infection, 3 analyses were performed on non-trauma acute surgery, and 3 analyses were performed on all acute settings. High prognostic value of RAPS in trauma patients was noticeable. The DOR of RAPS in predicting the in-hospital mortality of trauma patients was 431, while the value in sepsis/infection, non-trauma setting, and all acute settings was 6, 29, and 3, respectively (Table 4).

**Diagnostic value of mREMS in predicting the in-hospital mortality**

In the evaluation of the diagnostic value of mREMS in predicting the in-hospital mortality, 3 articles were included, which involved 13 separate analyses in terms of different cut-off points. The total number of patients in these 3 studies was 157749 patients, of which 12110 patients died. The cut-off points presented for this model in the studies varied between 3 and 14.

**Figure 4:**
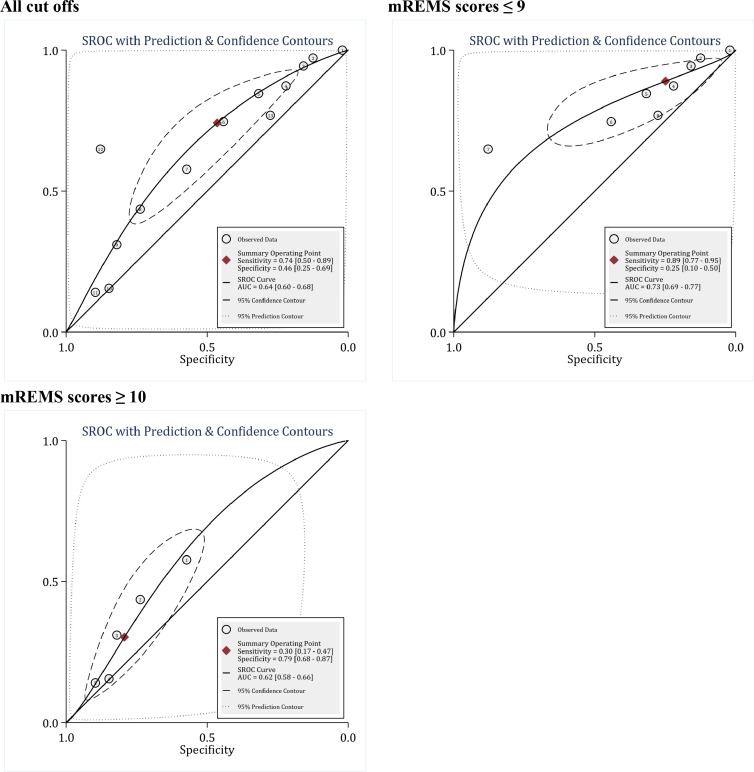
Prognostic value of modified Rapid Acute Physiology Score (mREMS) in prediction of in-hospital mortality. SROC: Summary receiver operating characteristics; AUC: area under the curve

The area under the ROC curve of mREMS in predicting the in-hospital mortality without considering the cut-off points was 0.64 (95% CI: 0.60 to 0.68). The sensitivity, specificity, and overall DOR of the mREMS model were lower than those of the REMS and RAPS scores, and were 0.74 (95% CI 0.50 to 0.89), 0.46 (95% CI: 0.25 to 0.69) and 3 (95% CI: 2 to 4), respectively (Figure 4-A and Table 6). However, there was significant heterogeneity between studies (I2 = 100.0%); Therefore, meta-regression analysis was performed. Meta-regression showed that the most important source of heterogeneity between studies in mREMS analyses was the use of different cut-off points, differences in study design (retrospective and prospective), and different patient settings (Table 2). Stratification of analyses based on these differences between studies caused a significant reduction in heterogeneity among studies to the point where I2 was zero in some subgroups. The findings were reported separately for these subgroups.

The first and the most important factor influencing the prognostic value of mREMS in predicting the in-hospital mortality was the different cut-off points used between studies. In this section, due to the small number of studies, mREMS cut-off points were divided into two groups: mREMS scores <10 and mREMS scores≥10. The area under the ROC curve of the mREMS model at the cut-off points of <10 and ≥10 was 0.73 (95% CI: 0.69 to 0.77) and 0.62 (95% CI: 0.58 to 0.66), respectively (Figure 4-B to 4- D). DOR was not significantly different between the two subgroups (3 versus 2, respectively) (Table 5).

All three studies included in this section were cohorts, so the type of study could not be the source of heterogeneity. Also, out of 13 analyses included in this section (from three studies), only 1 had a retrospective design analysis. Therefore, the difference in study design could not be the source of heterogeneity in examining the prognostic value of mREMS. Finally, it was found that 11 analyses of this section were performed on sepsis / infection patients, 1 analysis was performed on trauma patients, and 1 analysis was performed on all acute settings, which also showed that patients’ settings could not be a source of heterogeneity (Table 5).

The diagnostic value of WPS in predicting the in-hospital mortality

In the evaluation of the diagnostic value of WPS in predicting the in-hospital mortality, 5 articles were included, which involved 5 separate analyses. The total number of patients in these 5 studies was 10,771 patients, of whom 786 patients died. The cut-off points presented for this model in the studies varied between 3 and 6.

**Figure 5:**
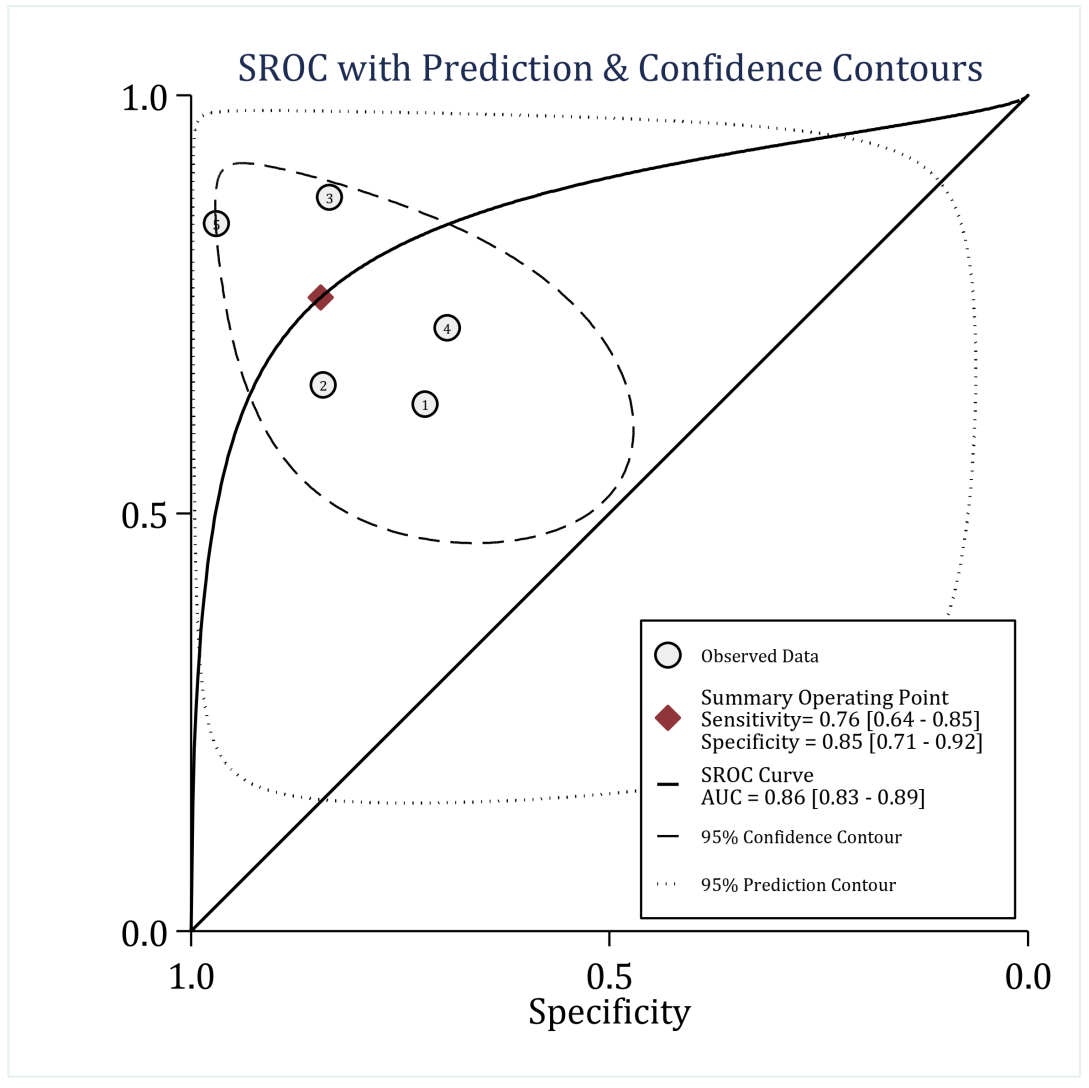
Prognostic value of Worthing Physiological Score (WPS) in prediction of in-hospital mortality. SROC: Summary receiver operating characteristics; AUC: area under the curve

The area under the ROC curve of WPS in predicting the in-hospital mortality without considering cut-off points was 0.86 (95% CI: 0.83 to 0.89). The sensitivity and specificity of this scoring model in predicting the in-hospital mortality were 0.76 (95% CI: 0.64 to 0.85) and 0.85 (95% CI: 0.71 to 0.92), respectively. Overall, the DOR of WPS was 17 (95% CI: 5 to 59) (Figure 5). In this section, there was significant heterogeneity between studies (I2 = 89.9%). Although meta-regression was performed in this part of the analysis, due to the the small number of studies, the origin of heterogeneity could not be identified and it was not possible to perform subgroup analysis (Table 2).

**3.3. Publication bias**

Deek’s funnel plot asymmetry test was used to examine the publication bias. This analysis showed that there was no evidence of publication bias in the relationship between REMS (p = 0.58), RAPS (p = 0.13), mREMS (p = 0.36), and WPS (p = 0.22) with in-hospital mortality. 

**3.4. Risk of bias assessment**

In the quality control of articles, it was found that 1 study had a high risk of bias in the patient selection section due to its case-control design. Additionally, the quality of the index test in 12 studies was unclear. The reason for this was the retrospective nature of the studies. In retrospective studies, physiological variables such as temperature, blood pressure, etc. are collected from patients' files; so, it is not clear how accurately these variables were recorded. Moreover, in the flow and timing section these 12 studies were high-risk, because data collection was done after the outcome (death) of patients was determined. In other items, the risk of bias and applicability were low (Table 6).

## 4. Discussion

Different studies have provided different cut-off points for the classification of patients at high risk of mortality in each of the physiological scoring systems of REMS, mREMS, RAPS, and WPS. Taking into account the uncertainty in the superiority of these physiological systems over each other in different patient settings, the present meta-analysis has, for the first time, collected the available evidence about the diagnostic value, sensitivity, and specificity of these physiological systems in acute patients and has tried to investigate the best cut-off point in each scoring system. The findings of the present study showed that RAPS, REMS and WPS, have a high predictive value for in-hospital mortality. A summary of the prognostic value of these models in identifying high-risk patients is presented in Table 7. Also, the value of these models in trauma patients is much higher than other patient settings. However, the evidence on the REMS model is greater than the other two models, and since the DOR of this model of identifying high-risk patients is very high, it is recommended to use REMS in emergency departments.

The RAPS model has been proposed for many years and its predictive value has been proven in some studies in adults (34). Years after the introduction of this model, REMS was introduced to increase the value of RAPS, in which patients' age and arterial oxygen saturation level were added to the variables in RAPS. Adding these two variables to RAPS increased its validity and REMS was proposed in the literature as an efficient model to classify damage severity (35, 36). Nevertheless, the findings of the present study show that REMS model is not more valuable than RAPS. Therefore, more studies are needed in this field to determine how much adding age and level of arterial oxygen saturation enhances the performance of these models.

Considering trauma patients as a separate group, it seems that using RAPS system is the best option to diagnose high-risk cases among these patients. This means that considering age and O2 sat, in addition to the RAPS components in the REMS criteria, lowers its diagnostic value. However, caution should be exercised in interpreting these results, because in our study, 2 studies (4 analyses) on the diagnostic value of RAPS in trauma patients were included, in one study, the sample population was children and the other was executed cross-sectionally. Moreover, in studies that defined the sample population as all acute patients, trauma patients were included, but it was not possible to separate these patients from the rest of the study population in our analyses; therefore, there is a possibility of a potential error that could not be eliminated due to the nature of this study as a systematic review, and further and more comprehensive studies are needed to investigate this issue.

Various scoring systems have been proposed to classify the severity of injury. These systems include physiological, anatomical, and combined scores as well as specialized trauma scoring systems (37). Each of these systems have different limitations and advantages; but the scoring system that can be used in acute conditions should have few variables and be easily calculated. Almost all scoring systems have a Glasgow Coma Scale (GCS) awareness level. In addition to GCS, these scoring models use physiological criteria such as body temperature, respiration rate, blood pressure, and heart rate to determine the severity of the injury. Nonetheless, the question is whether adding these physiological criteria to GCS would sufficiently and significantly lead to a better and more accurate diagnosis of injury severity. To answer this question, an article was conducted on 1,702 patients, and showed that the predictive value of GCS is similar to physiological scoring models. The study concluded that GCS is the best model for predicting patient mortality as it is easier than physiological models to calculate and has fewer variables. Also, its predictive value is not significantly different from these models (38). Therefore, it is suggested that more studies be conducted to compare the value of physiological models with GCS.

This study, like other retrospective studies, had its limitations. First, the quality of recording the clinical characteristics of patients in the emergency department could not be assessed. Also, the number of studies performed in each acute setting was different and limited. 

This study has had many strengths. The study population was a total of 737,351 patients, which is considerable; in addition, four physiological systems designed for evaluation of acute patients in emergency settings, including REMS, RAPS, mREMS and WPS, have been simultaneously studied in patients with trauma, sepsis, acute conditions, and non-traumatic acute conditions. It should be noted that most of the studies included were prospective cohorts.

**Table 1:** Characteristics of included studies

**Author; year; country**	**Study type**	**Sample size**	**N male**	**N mortality**	**Setting of patients**	**Age**	**Timing (hr)**	**Score**	**Cut off**
Badrinath; 2018; India (39)	PCS	193	125	108	Sepsis	57.2 ± 15.3	0	REMS	5
Chang; 2018; Taiwan (40)	CC	152	48	8	Renal abscess	54 (41-65)	0	RAPS; REMS	4; 6
Crowe; 2010; USA (41)	PCS	216	109	71	Sepsis	71 (59-81)	0	mREMS	3 to 14
Crowe; 2020; USA (42)	RCS	484865	213058	7114	All acute setting patients	61 (43-76)	0	REMS	3 to 11
Demircan; 2018; Turkey (43)	PCS	1106	528	173	All acute setting patients	77 ±7	0	REMS	9.5
Duckitt; 2007; UK (44)	PCS	4286	2024	355	All acute setting patients	17-106	0	WPS	3
Dundar; 2015; Turkey (45)	PCS	939	507	73	Elderly patients without history of trauma or resuscitation	74 ± 11	0.16	REMS	8
Hung; 2017; Taiwan (46)	RCS	114	77	14	Sepsis and splenic abscess	55 (43-72)	0	RAPS; RAPS	4; 7
Kuo; 2013; Taiwan (47)	RCS	171	95	43	Sepsis	63 ± 12	24	REMS	8
Lee; 2020; Korea (48)	RCS	27173	15663	2057	All acute setting patients	64 (50-75)	0	REMS	3
Liu; 2020; China (49)	RCS	673	341	121	COVID-19	61 (50-69)	0	REMS; WPS; mREMS	6; 6; 9
Nakhjavan-shahraki; 2017-a; Iran (50)	Cross-sectional	2148	1623	123	Trauma	39 ± 17	0	RAPS; REMS	2; 3
Nakhjavan-shahraki; 2017-b; Iran (51)	Cross-sectional	2148	1623	123	Trauma	39 ± 17	0	WPS	4
Nakhjavan-shahraki; 2017-c; Iran (52)	PCS	814	605	26	Trauma	11 ±5	0	RAPS; REMS; WPS	3; 3; 6
Olsson; 2004; Sweden (53)	PCS	11751	5688	285	Non-surgical acute setting	62 ± 21	0.33	RAPS; REMS	2-4; 3-11
Park; 2017; Korea (54)	RCS	6905	4298	212	Trauma	57 ±18	0	REMS	7
Park; 2019; Korea (55)	RCS	582	420	87	Trauma	59 (46-78)	0	REMS	8
Seak; 2017; Taiwan (56)	RCS	66	36	38	GI complication	69 ± 17	0	RAPS; REMS	4; 11
Sewalt; 2019; England (57)	RCS	154476	82979	11882	Trauma	66 (47-83)	0	mREMS	3
Sharma; 2013; United States (58)	PCS	241	145	34	Bacteremia	59 ± 18	0	REMS	6
Swain; 2020; India (59)	PCS	100	51	24	sepsis	49 ± 14	0	REMS	7
Söyüncü; 2011; Turkey (60)	PCS	30	16	3	Intoxication	30 ± 14	0	RAPS; REMS	8; 9
Wei; 2019; China (61)	RCS	39977	19131	213	non-trauma patients	44 ± 18	0	RAPS; REMS	7; 8
Yang; 2017; China (62)	PCS	123	62	31	Severe fever with thrombocytopenia syndrome	NR	0	REMS	9.5
Yüksel Gök; 2019; Turkey (63)	RCS	250	144	54	All acute setting patients	58 ± 21	0	REMS; WPSS	6; 5

RCS: Retrospective cohort study; PCS: Prospective cohort study; CC: Case-control study; GI: gastrointestinal; REMS: Rapid Emergency Medicine Score; RAPS: Rapid Acute Physiology Score; WPS: Worthing Physiological Score; mREMS: modified REMS.

**Table 2:** Meta-regression for assessment of source of heterogeneity in values of REMS, RAPS, mREMS, and WPS scores in prediction of in-hospital mortality

**Parameter**	**REMS score**					**RAPS score**					**mREMS**					**WPS**			
	**n**	**LRTChi** ^2^	**I** ^2^	**P**		**n**	**LRTChi** ^2^	**I** ^2^	**P**		**n**	**LRTChi** ^2^	**I** ^2^	**P**		**n**	**LRTChi** ^2^	**I** ^2^	**P**
**Study type**																			
Cohort	36	*ref.*	*ref.*	*ref.*		8	*ref.*	*ref.*	*ref.*		13	*NA*	*NA*	*NA*		4	*ref.*	*ref.*	*ref.*	
Case-control	1	0.15	0.0	0.93		1	0.93	0.0	0.63		-	-	-	-					
Cross sectional	1	4.45	55	0.11		1	0.37	0.0	0.83		-	-	-	-		1	9.08	78	0.01
**Study design**																			
Prospective	19	*ref.*	*ref.*	*ref.*		6	*ref.*	*ref.*	*ref.*		12	*ref.*	*ref.*	*ref.*		4	*ref.*	*ref.*	*ref.*
Retrospective	19	1.62	0.0	0.44		4	0.45	0.0	0.80		1	1.63	0.0	0.44		1	1.39	0.0	0.5
**Setting of patients**																			
Trauma	4	*ref.*	*ref.*	*ref.*		2	*ref.*	*ref.*	*ref.*		1	*ref.*	*ref.*	*ref.*		2	*ref.*	*ref.*	*ref.*
Sepsis/infection	9	0.03	0.0	0.98		2	0.66	0.0	0.72		11	1.77	0.0	0.41		-	-	-	-
Non-trauma acute settings	4	4.22	53	0.12		3	1.62	0.0	0.44		1	17.88	89	<0.001		-	-	-	-
All acute settings	21	11.57	83	<0.001		3	6.49	69	0.04		-	-	-	-		3	14.44	86	<0.001
**Cut off**																			
3	5	*ref.*	*ref.*	*ref.*		6*	*ref.*	*ref.*	*ref.*		2	*ref.*	*ref.*	*ref.*		1	*ref.*	*ref.*	*ref.*
4	2	6.56	69	0.04		4	0.43	0.0	0.81		-	-	-	-		1	9.08	78	0.01
5	3	1.77	0.0	0.41		-	-	-	-		1	1.97	0.0	0.37		1	1.39	0.0	0.50
6	7	3.59	44	0.17		-	-	-	-		1	0.99	0.0	0.61		2	8.2	76	0.02
7	5	0.22	0.0	0.90		2^#^	2.67	25	0.26		1	0.55	0.0	0.76		-	-	-	-
8	6	1.69	0.0	0.43		-	-	-	-		1	0.14	0.0	0.93		-	-	-	-
9	3	1.46	0.0	0.48		-	-	-	-		2	7.02	72	0.03		-	-	-	-
9.5	2	3.43	42	0.18		-	-	-	-		-	-	-	-		-	-	-	-
10	2	4.29	53	0.12		-	-	-	-		1	0.25	0.0	0.88		-	-	-	-
11	3	7.75	74	0.02		-	-	-	-		1	0.53	0.0	0.77		-	-	-	-
12	-	-	-	-		-	-	-	-		1	1.09	0.0	0.58		-	-	-	-
13	-	-	-	-		-	-	-	-		1	3.07	35	0.22		-	-	-	-
14	-	-	-	-		-	-	-	-		1	2.74	27	0.25		-	-	-	-

*, Scores 2 and 3; #, scores 7 and 8, n: Number of analyses. REMS: Rapid Emergency Medicine Score; RAPS: Rapid Acute Physiology Score; WPS: Worthing Physiological Score; mREMS: modified REMS.

**Table 3:** Subgroup analysis for value of Rapid Emergency Medicine Score (REMS) in prediction of in-hospital mortality

**Parameter**	**No. analyses**	**AUC**	**Sensitivity**	**Specificity**	**PLR**	**NLR**	**DOR**
**REMS score**							
3 to 5	10	0.87 [0.84, 0.90]	0.96 [0.89, 0.99]	0.52 [0.34, 0.70]	2.0 [1.4, 2.9]	0.07 [0.03, 0.20]	27 [8, 84]
6 to 8	18	0.83 [0.79, 0.86]	0.80 [0.76, 0.84]	0.70 [0.63, 0.75]	2.6 [2.2, 3.2]	0.28 [0.23, 0.34]	9 [7, 13]
9 to 11	10	0.80 [0.76, 0.83]	0.55 [0.42, 0.67]	0.86 [0.79, 0.90]	3.8 [2.8, 5.1]	0.53 [0.42, 0.68]	7 [5, 11]
**Study type**							
Cohort	36	0.83 [0.79, 0.86]	0.82 [0.74, 0.87]	0.71 [0.63, 0.77]	2.8 [2.3, 3.4]	0.26 [0.19, 0.35]	11 [8, 15]
Case-control	1	NA	NA	NA	NA	NA	NA
Cross sectional	1	NA	NA	NA	NA	NA	NA
**Study design**							
Prospective	19	0.84 [0.81, 0.87]	0.86 [0.74, 0.93]	0.71 [0.60, 0.80]	3.0 [2.2, 4.0]	0.19 [0.11, 0.35]	15 [8, 29]
Retrospective	19	0.82 [0.78, 0.85]	0.79 [0.70, 0.86]	0.70 [0.60, 0.79]	2.7 [2.1, 3.4]	0.29 [0.22, 0.40]	9 [7, 13]
**Setting of patients**							
Trauma	4	0.92 [0.89 - 0.94]	0.96 [0.53, 1.00]	0.84 [0.73, 0.90]	5.8 [3.1, 10.7]	0.05 [0.00, 0.95]	112 [4, 3331]
Sepsis/infection	9	0.82 [0.79, 0.85	0.79 [0.75, 0.83]	0.71 [0.63, 0.78]	2.8 [2.1, 3.5]	0.29 [0.24, 0.35]	9 [7, 14]
Non-trauma settings	4	0.88 [0.85, 0.91]	0.77 [0.68, 0.84]	0.86 [0.80, 0.90]	5.4 [3.4, 8.6]	0.27 [0.18, 0.40]	20 [9, 46]
All acute settings	21	0.79 [0.75, 0.82]	0.81 [0.68, 0.90]	0.65 [0.52, 0.75]	2.3 [1.8, 2.9]	0.29 [0.19, 0.44]	8 [6, 11]

Data are presented as value [95% confidence interval].

AUC: Area under the curve; DOR: Diagnostic odds ratio; NA: Not applicable since there is a small number of studies; NLR: Negative likelihood ratio; PLR: Positive likelihood ratio.

**Table 4:** Subgroup analyses for value of Rapid Acute Physiology Score (RAPS) in prediction of in-hospital mortality

**Parameter**	**No. analyses**	**AUC**	**Sensitivity**	**Specificity**	**PLR**	**NLR**	**DOR**
**RAPS score**							
2 to 3	6	0.93 [0.90 - 0.95]	0.89 [0.66, 0.97]	0.83 [0.66, 0.93]	5.4 [2.2, 13.3]	0.13 [0.03, 0.52]	42 [5, 386]
4	4	0.81 [0.77 - 0.84]	0.72 [0.55, 0.84]	0.78 [0.73, 0.82]	3.2 [2.7, 3.8]	0.36 [0.22, 0.59]	9 [5, 16]
7 to 8	2	0.94 [0.91 - 0.96]	0.87 [0.32, 0.99]	0.91 [0.86, 0.94]	9.8 [6.8, 14.0]	0.14 [0.01, 1.41]	69 [6, 782]
**Study type**							
Cohort	8	0.85 [0.82 - 0.88]	0.73 [0.49, 0.88]	0.82 [0.68, 0.91]	4.1 [2.0, 8.3]	0.33 [0.15, 0.73]	12 [3, 49]
Case-control	1	NA	NA	NA	NA	NA	NA
Cross sectional	1	NA	NA	NA	NA	NA	NA
**Study design**							
Prospective	6	0.87 [0.84 - 0.90]	0.81 [0.49, 0.95]	0.80 [0.63, 0.90]	4.1 [1.8, 9.4]	0.24 [0.07, 0.86]	17 [2, 128]
Retrospective	4	0.85 [0.81 - 0.88]	0.73 [0.54, 0.87]	0.82 [0.68, 0.91]	4.1 [2.5, 6.8]	0.33 [0.19, 0.55]	13 [7, 22]
**Setting of patients**							
Trauma	4	0.97 [0.95 - 0.98]	0.98 [0.66, 1.00]	0.91 [0.83, 0.95]	10.5 [5.5, 20.1]	0.02 [0.00, 0.53]	431 [12, 15271]
Sepsis/infection	2	0.76 [0.72 - 0.80]	0.68 [0.60, 0.75]	0.73 [0.71, 0.74]	2.5 [2.2, 2.8]	0.48 [0.34, 0.68]	6 [4, 8]
Non-trauma settings	3	0.92 [0.89 - 0.94]	0.79 [0.67, 0.87]	0.89 [0.85, 0.92]	7.1 [5.7, 8.8]	0.24 [0.15, 0.38]	29 [18, 47]
All acute settings	3	0.63 [0.59 - 0.68]	0.55 [0.49, 0.61]	0.68 [0.59, 0.75]	1.7 [1.5, 2.0]	0.66 [0.64, 0.68]	3 [2, 3]

Data are presented as value [95% confidence interval]. AUC: Area under the curve; DOR: Diagnostic odds ratio; NA: Not applicable since there are limited/is a small number of studies; NLR: Negative likelihood ratio; PLR: Positive likelihood ratio.

**Table 5:** Subgroup analyses for value of modified Rapid Acute Physiology Score (mREMS) in prediction of in-hospital mortality

**Parameter**	**No. analyses**	**AUC**	**Sensitivity**	**Specificity**	**PLR**	**NLR**	**DOR**
**mREMS score**							
3 to 9	8	0.73 [0.69 - 0.77]	0.89 [0.77, 0.95]	0.25 [0.10, 0.50]	1.2 [1.0, 1.5]	0.44 [0.27, 0.72]	3 [1, 5]
10 to 14	5	0.62 [0.58 - 0.66]	0.30 [0.17, 0.47]	0.79 [0.68, 0.87]	1.5 [1.2, 1.8]	0.88 [0.78, 1.00]	2 [1, 2]
**Study type**							
Cohort	13	0.64 [0.60 - 0.68]	0.74 [0.50, 0.89]	0.46 [0.25, 0.6	1.4 [1.1, 1.7]	0.56 [0.38, 0.82]	2 [2, 4]
Case-control	0	---	---	---	---	---	---
Cross sectional	0	---	---	---	---	---	---
**Study design**							
Prospective	12	0.65 [0.61 - 0.69]	0.75 [0.47, 0.91]	0.48 [0.25, 0.72]	1.4 [1.1, 1.9]	0.52 [0.33, 0.82]	3 [2, 4]
Retrospective	1	NA	NA	NA	NA	NA	NA
**Setting of patients**							
Trauma	1	NA	NA	NA	NA	NA	NA
Sepsis/infection	11	0.59 [0.55 - 0.63]	0.73 [0.47, 0.89]	0.41 [0.21, 0.65]	1.2 [1.1, 1.4]	0.65 [0.48, 0.88]	2 [1, 3]
Non-trauma settings	0	NA	NA	NA	NA	NA	NA
All acute settings	1	NA	NA	NA	NA	NA	NA

Data are presented as value [95% confidence interval].

AUC: Area under the curve; DOR: Diagnostic odds ratio; NA: Not applicable since there is a small number of studies; NLR: Negative likelihood ratio; PLR: Positive likelihood ratio.

**Table 6:** Risk of bias assessment among included studies

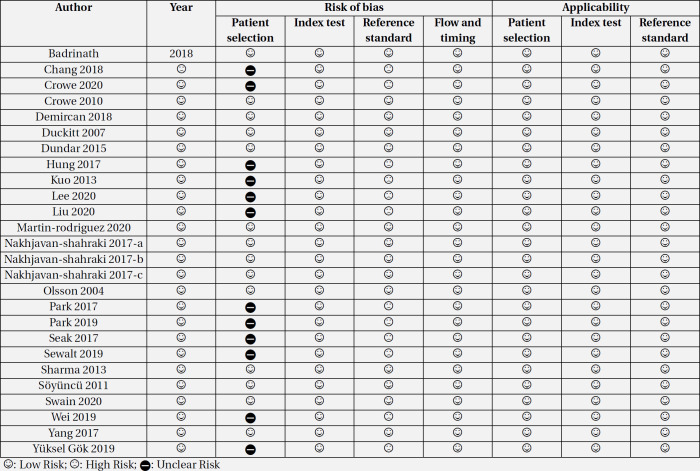

**Table 7:** Summary of prognostic performance of physiologic scores

Score	Number of studies	SROC (95% CI)	Sensitivity	Specificity	DOR
REMS	21	0.83 (0.79 to 0.86)	0.83 (0.75 to 0.88)	0.71 (0.63 to 0.77)	11 (8 to 16)
RAPS	8	0.89 (0.86 to 0.92)	0.82 (0.63 to 0.92)	0.83 (0.74 to 0.90)	13 (4 to 41)
mREMS	3	0.64 (0.60 to 0.68)	0.74 (0.50 to 0.89)	0.46 (0.25 to 0.69)	3 (2 to 4)
WPS	5	0.86 (0.83 to 0.89)	0.76 (0.64 to 0.85)	0.85 (0.71 to 0.92)	17 (5 to 59)

DOR: Diagnostic odds ratio; mREMS: modified REMS; RAPS: Rapid acute physiology score; REMS: Rapid emergency medicine score; SROC: Summary receiver operating characteristics; WPS: Worthing physiological score

**Figure 6:**
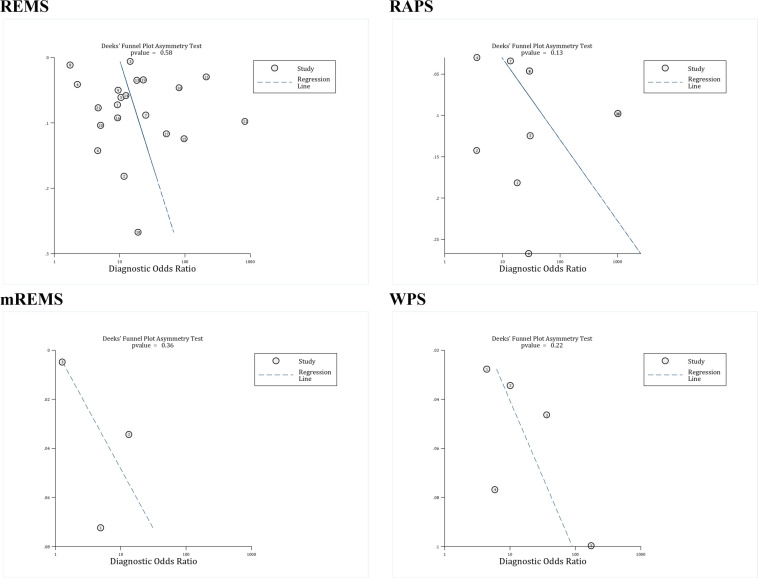
Publication bias assessment among included studies based on assessed scores. There is no evidence of publication bias among studies. RAPS: Rapid acute physiology score; REMS: Rapid emergency medicine score; mREMS: modified REMS; WPS: Worthing physiological score

## 5. Conclusion:

The findings of the present study showed that RAPS, REMS and WPS, have a high predictive value in in-hospital mortality. Also, the value of these models in trauma patients is much higher than other patients. However, the number of articles on the REMS model is more than the other two models, and since the DOR of this model is high in identifying high-risk patients, it is recommended to use REMS in acute conditions to identify high-risk patients.

## 6. Declaration:

### 6.1. Acknowledgment

None.

### 6.2. Funding

This study was funded and supported by a grant from Iran university of medical sciences. 

### 6.3. Competing of interest

The authors declared no conflict of interest. 

### 6.4. Authors’ contribution

Study design: MY, MH, AT

Data gathering: MY, AT, AMN, MIMG, FM

Analysis: MH, MKG, AZSK

Interpretation: All authors

Drafting: AT, MY

Revising: All authors

## References

[1] Meheš M, Abdullah F (2011). Global Surgery and Public Health: A New Paradigm. Archives of Surgery.

[2] Saadat S, Yousefifard M, Asady H, Jafari AM, Fayaz M, Hosseini M (2015). The most important causes of death in Iranian population; a Retrospective Cohort Study. Emergency.

[3] Mathers CD, Loncar D (2006). Projections of Global Mortality and Burden of Disease from 2002 to 2030. PLoS Med.

[4] Mommsen P, Zeckey C, Andruszkow H, Weidemann J, Frömke C, Puljic P (2012). Comparison of Different Thoracic Trauma Scoring Systems in Regards to Prediction of Post-Traumatic Complications and Outcome in Blunt Chest Trauma. Journal of Surgical Research.

[5] Staff T, Eken T, Wik L, Røislien J, Søvik S (2013). Physiologic, demographic and mechanistic factors predicting New Injury Severity Score (NISS) in motor vehicle accident victims. Injury.

[6] Yu S, Leung S, Heo M, Soto GJ, Shah RT, Gunda S (2014). Comparison of risk prediction scoring systems for ward patients: a retrospective nested case-control study. Critical care (London, England).

[7] de Pennington J, Laurenson J, Lebus C, Sihota S, Smith P (2005). Evaluation of early warning systems on a medical admissions unit. Journal of the Intensive Care Society.

[8] Goldhill D (2005). Preventing surgical deaths: critical care and intensive care outreach services in the postoperative period. British journal of anaesthesia.

[9] Morgan R, Williams F, Wright M (1997). An early warning scoring system for detecting developing critical illness. Clin Intensive Care.

[10] Williams E, Subbe C, Gemmell L, Morgan R, Park G, McElligot M (2003). Outreach critical care—cash for no questions?. British journal of anaesthesia.

[11] Duckitt RW, Buxton-Thomas R, Walker J, Cheek E, Bewick V, Venn R (2007). Worthing physiological scoring system: derivation and validation of a physiological early-warning system for medical admissions An observational, population-based single-centre study† ‡. British Journal of Anaesthesia.

[12] Huang J, Xuan D, Li X, Ma L, Zhou Y, Zou H (2017). The value of APACHE II in predicting mortality after paraquat poisoning in Chinese and Korean population: A systematic review and meta-analysis. Medicine (Baltimore).

[13] Hamilton F, Arnold D, Baird A, Albur M, Whiting P (2018). Early Warning Scores do not accurately predict mortality in sepsis: A meta-analysis and systematic review of the literature. Journal of Infection.

[14] Manoochehry S, Vafabin M, Bitaraf S, Amiri A (2019). A comparison between the ability of revised trauma score and Kampala trauma score in predicting mortality; a meta-analysis. Archives of academic emergency medicine.

[15] Zhang K, Zhang G (2020). An updated meta-analysis of modified early warning scores in patients with sepsis outside intensive care unit. The Journal of infection.

[16] Nakhjavan-Shahraki B, Baikpour M, Yousefifard M, Nikseresht ZS, Abiri S, Razaz JM (2017). Rapid Acute Physiology Score versus Rapid Emergency Medicine Score in Trauma Outcome Prediction; a Comparative Study. Emergency.

[17] Yousefifard M, Nakhjavan-Shahraki B, Sarveazad A, Safari S, Hosseini M (2017). The relationship of hemodynamic parameters with 6-month mortality in trauma patients; a prospective cohort study. Journal of Medical Physiology.

[18] Nakhjavan-Shahraki B, Yousefifard M, Hajighanbari M, Oraii A, Safari S, Hosseini M (2017). Pediatric Emergency Care Applied Research Network (PECARN) prediction rules in identifying high risk children with mild traumatic brain injury. European journal of trauma and emergency surgery.

[19] Shojaee M, Faridaalaee G, Yousefifard M, Yaseri M, Arhami Dolatabadi A, Sabzghabaei A (2014). New scoring system for intra-abdominal injury diagnosis after blunt trauma. Chin J Traumatol.

[20] Stroup DF, Berlin JA, Morton SC, Olkin I, Williamson GD, Rennie D (2000). Meta-analysis of observational studies in epidemiology: a proposal for reporting. Jama..

[21] Ebrahimi A, Yousefifard M, Kazemi HM, Rasouli HR, Asady H, Jafari AM (2014). Diagnostic accuracy of chest ultrasonography versus chest radiography for identification of pneumothorax: a systematic review and meta-analysis. Tanaffos.

[22] Hassanzadeh‐Rad A, Yousefifard M, Katal S, Asady H, Fard‐Esfahani A, Moghadas Jafari A (2016). The value of 18F‐fluorodeoxyglucose positron emission tomography for prediction of treatment response in gastrointestinal stromal tumors: a systematic review and meta‐analysis. Journal of gastroenterology and hepatology.

[23] Higgins JP, Green S (2011). Cochrane handbook for systematic reviews of interventions.

[24] Rahimi-Movaghar V, Yousefifard M, Ghelichkhani P, Baikpour M, Tafakhori A, Asady H (2015). Application of ultrasonography and radiography in detection of hemothorax: a systematic review and meta-analysis. Emergency.

[25] Yousefifard M, Baikpour M, Ghelichkhani P, Asady H, Darafarin A, Esfahani MRA (2016). Comparison of Ultrasonography and Radiography in Detection of Thoracic Bone Fractures; a Systematic Review and Meta-Analysis. Emergency.

[26] Izadi A, Yousefifard M, Nakhjavan-Shahraki B, Baikpour M, Mirzay Razaz J, Ataei N (2016). Value of Plasma/Serum Neutrophil Gelatinase-Associated Lipocalin in Detection of Pediatric Acute Kidney Injury; a Systematic Review and Meta-Analysis. International Journal of Pediatrics.

[27] Izadi A, Yousefifard M, Nakhjavan-Shahraki B, Baikpour M, Mirzay Razaz J, Hosseini M (2016). Diagnostic Value of Urinary Neutrophil Gelatinase-Associated Lipocalin (NGAL) in Detection of Pediatric Acute Kidney Injury; a Systematic Review and Meta-Analysis. International Journal of Pediatrics.

[28] Rahimi-Movaghar V, Yousefifard M, Ghelichkhani P, Baikpour M, Tafakhori A, Asady H (2016). Application of Ultrasonography and Radiography in Detection of Hemothorax: a Systematic Review and Meta-Analysis. EMERGENCY-An Academic Emergency Medicine Journal.

[29] Yousefifard M, Rahimi-Movaghar V, Nasirinezhad F, Baikpour M, Safari S, Saadat S (2016). Neural stem/progenitor cell transplantation for spinal cord injury treatment; A systematic review and meta-analysis. Neuroscience..

[30] Hosseini M, Yousefifard M, Aziznejad H, Nasirinezhad F (2015). The Effect of Bone Marrow–Derived Mesenchymal Stem Cell Transplantation on Allodynia and Hyperalgesia in Neuropathic Animals: A Systematic Review with Meta-Analysis. Biology of Blood and Marrow Transplantation.

[31] Moher D, Liberati A, Tetzlaff J, Altman DG (2009). Preferred reporting items for systematic reviews and meta-analyses: the PRISMA statement. Annals of internal medicine.

[32] Whiting PF, Rutjes AW, Westwood ME, Mallett S, Deeks JJ, Reitsma JB (2011). QUADAS-2: a revised tool for the quality assessment of diagnostic accuracy studies. Ann Intern Med.

[33] Jin Z-C, Wu C, Zhou X-H, He J (2014). A modified regression method to test publication bias in meta-analyses with binary outcomes. BMC Medical Research Methodology..

[34] Bertollo S, Rodenberg H (1994). Correlation of the RTS and RAPS in rotor-wing prehospital care. Air medical journal.

[35] Olsson T, Lind L (2003). Comparison of the Rapid Emergency Medicine Score and APACHE II in Nonsurgical Emergency Department Patients. Academic Emergency Medicine.

[36] Olsson T, Terént A, Lind L (2004). Rapid Emergency Medicine Score: a new prognostic tool for in‐hospital mortality in nonsurgical emergency department patients. Journal of internal medicine.

[37] Lefering R (2012). Trauma scoring systems. Current opinion in critical care.

[38] Yousefifard M, Shahsavarinia K, Faridaalee G, Dinpanah H, Ahmadi S, Safari S (2020). Comparison of Glasgow Coma Scale with Physiologic Scoring Scales in Prediction of In-Hospital Outcome of Trauma Patients; a Diagnostic Accuracy Study. Advanced Journal of Emergency Medicine.

[39] Badrinath K, Shekhar M, Sreelakshmi M, Srinivasan M, Thunga G, Nair S (2018). Comparison of various severity assessment scoring systems in patients with sepsis in a tertiary care teaching hospital. Indian Journal of Critical Care Medicine.

[40] Chang SH, Hsieh CH, Weng YM, Hsieh MS, Goh ZNL, Chen HY (2018). Performance assessment of the mortality in emergency department sepsis score, modified early warning score, rapid emergency medicine score, and rapid acute physiology score in predicting survival outcomes of adult renal abscess patients in the emergency department. BioMed Research International.

[41] Crowe CA, Kulstad EB, Mistry CD, Kulstad CE (2010). Comparison of severity of illness scoring systems in the prediction of hospital mortality in severe sepsis and septic shock. Journal of emergencies, trauma, and shock.

[42] Crowe RP, Bourn S, Fernandez AR, Myers JB (2020). Initial Prehospital Rapid Emergency Medicine Score (REMS) as a Predictor of Patient Outcomes Prehospital emergency care : official journal of the National Association of EMS Physicians and the National Association of State EMS Directors.

[43] Demircan S, Ergin M, Tanriverdi F, Elgörmüş ÇS, Kurtoğlu Çelik G, Özhasenekler A (2018). Combination of lactate with modified early warning score and rapid emergency medicine score in geriatric patients admitted to emergency department to predict 28-day mortality. Turk Geriatri Dergisi.

[44] Duckitt RW, Buxton-Thomas R, Walker J, Cheek E, Bewick V, Venn R (2007). Worthing physiological scoring system: derivation and validation of a physiological early-warning system for medical admissions An observational, population-based single-centre study. Br J Anaesth.

[45] Dundar ZD, Karamercan MA, Ergin M, Colak T, Tuncar A, Ayranci K (2015). Rapid Emergency Medicine Score and HOTEL Score in Geriatric Patients Admitted to the Emergency Department. International Journal of Gerontology.

[46] Hung SK, Ng CJ, Kuo CF, Goh ZNL, Huang LH, Li CH (2017). Comparison of the Mortality in Emergency Department Sepsis Score, Modified Early Warning Score, Rapid Emergency Medicine Score and Rapid Acute Physiology Score for predicting the outcomes of adult splenic abscess patients in the emergency department. PloS one.

[47] Kuo SH, Tsai CF, Li CR, Tsai SJ, Chao WN, Chan KS (2013). Rapid Emergency Medicine Score as a main predictor of mortality in Vibrio vulnificus-related patients. The American journal of emergency medicine.

[48] Lee SB, Kim DH, Kim T, Kang C, Lee SH, Jeong JH (2020). Emergency Department Triage Early Warning Score (TREWS) predicts in-hospital mortality in the emergency department. The American journal of emergency medicine.

[49] Liu FY, Sun XL, Zhang Y, Ge L, Wang J, Liang X (2020). Evaluation of the Risk Prediction Tools for Patients With Coronavirus Disease 2019 in Wuhan, China: A Single-Centered, Retrospective, Observational Study. Critical care medicine.

[50] Nakhjavan-Shahraki B, Baikpour M, Yousefifard M, Nikseresht ZS, Abiri S, Mirzay Razaz J (2017-a). Rapid Acute Physiology Score versus Rapid Emergency Medicine Score in Trauma Outcome Prediction; a Comparative Study. Emergency (Tehran, Iran).

[51] Nakhjavan-Shahraki B, Yousefifard M, Faridaalaee G, Shahsavari K, Oraii A, Hajighanbari MJ (2017-c). Performance of physiology scoring systems in prediction of in-hospital mortality of traumatic children: A prospective observational study. Journal of clinical orthopaedics and trauma.

[52] Nakhjavan-Shahraki B, Yousefifard M, Hajighanbari MJ, Karimi P, Baikpour M, Mirzay Razaz J (2017-b). Worthing Physiological Score vs Revised Trauma Score in Outcome Prediction of Trauma patients; a Comparative Study. Emergency (Tehran, Iran).

[53] Olsson T, Terent A, Lind L (2004). Rapid Emergency Medicine score: a new prognostic tool for in-hospital mortality in nonsurgical emergency department patients. J Intern Med.

[54] Park HO, Kim JW, Kim SH, Moon SH, Byun JH, Kim KN (2017). Usability verification of the Emergency Trauma Score (EMTRAS) and Rapid Emergency Medicine Score (REMS) in patients with trauma: A retrospective cohort study. Medicine (Baltimore).

[55] Park HO, Choi JY, Jang IS, Kim JD, Choi JW, Lee CE (2019). Assessment of the Initial Risk Factors for Mortality among Patients with Severe Trauma on Admission to the Emergency Department. The Korean journal of thoracic and cardiovascular surgery.

[56] Seak CJ, Yen DHT, Ng CJ, Wong YC, Hsu KH, Seak JCY (2017). Rapid Emergency Medicine Score: A novel prognostic tool for predicting the outcomes of adult patients with hepatic portal venous gas in the emergency department. PloS one.

[57] Sewalt CA, Venema E, Wiegers EJA, Lecky FE, Schuit SCE, den Hartog D (2020). Trauma models to identify major trauma and mortality in the prehospital setting. The British journal of surgery.

[58] Sharma M, Szpunar S, Khatib R (2013). Validating severity of illness scoring systems in the prediction of outcomes in Staphylococcus aureus bacteremia. The American journal of the medical sciences.

[59] Swain SK, Patra JK, Kumar SR, Choudhury A, Padhi PK, Thatoi PK (2020). Prediction of Mortality in Sepsis using Rapid Emergency Medicine Score: A Cohort Study. J Clin Diagn Res.

[60] Söyüncü S, Bektaş F (2011). Comparison of the scoring systems for predicting mortality in intoxicated patients hospitalized to the icu: A prospective observational study. Erciyes Tip Dergisi.

[61] Wei XJ, Ma HL, Liu RN, Zhao Y (2019). Comparing the effectiveness of three scoring systems in predicting adult patient outcomes in the emergency department. Medicine (Baltimore).

[62] Yang B, Wang X, Li Y, Wu A, Liu Q, Lu Y (2017). A newly established severity scoring system in predicting the prognosis of patients with severe fever with thrombocytopenia syndrome. Tohoku Journal of Experimental Medicine.

[63] Gök RGY, Gök A, Bulut M (2019). Assessing prognosis with modified early warning score, rapid emergency medicine score and worthing physiological scoring system in patients admitted to intensive care unit from emergency department. International emergency nursing.

